# Record of the circumstances of falls in the community: perspective in the Iberian Peninsula^1^

**DOI:** 10.1590/1518-8345.2373.2977

**Published:** 2018-07-16

**Authors:** Maria de Fátima Araújo, Nilza Nogueira Caldevilla, Candida Maciel, Felicidade Malheiro, María Aurora Rodríguez-Borrego, Pablo Jesús López-Soto

**Affiliations:** 2 PhD, Adjunct Professor, Escola Superior de Enfermagem do Porto (ESEP), Porto, Portugal.; 3 MSc, RN, Unidade de Saúde Familiar Arca d’Água, Porto, Portugal.; 4 General and Family Medicine Specialist, MD, Unidade de Saúde Familiar Arca d’Água, Porto, Portugal.; 5 PhD, Full Professor, Instituto Maimónides de Investigación Biomédica de Córdoba, Hospital Universitario Reina Sofía, Universidad de Córdoba, Córdoba, Spain.; 6 PhD, Assistant Professor, Instituto Maimónides de Investigación Biomédica de Córdoba, Hospital Universitario Reina Sofía, Universidad de Córdoba, Córdoba, Spain.

**Keywords:** Accidental falls, Community Health Workers, Community Health Nursing, Accident Prevention, Risk Assessment, Health Promotion

## Abstract

**Objective:**

to determine the diagnosis of the situation regarding documentation of falls and risk of falls in people older than 75 years in basic health units in Spain and Portugal.

**Method:**

mixed exploratory study in two stages: (i) quantitative descriptive of randomly selected fall records produced in one year (597 records; 197 Spanish and 400 Portuguese); and (ii) qualitative, with the purpose of knowing the perception of health professionals employing semi-structured interviews (72 professionals, 16 Spanish and 56 Portuguese). The study areas were two basic health units in southern Spain and northern Portugal.

**Results:**

in the fall records, the number of women was higher. The presence of fall was associated with the variables age, presence of dementia, osteoarticular disease, previous falls and consumption of antivertiginous medication. Health professionals perceived an absence of risk assessment instruments, as well as lack of prevention programs and lack of awareness of this event.

**Conclusion:**

falls are perceived as an area of ​​priority attention for health professionals. Nonetheless, there is a lack of adherence to the registration of falls and risk assessment, due to organizational, logistical and motivational problems.

## Introduction

In an aging population, it is vitally important to develop public health policies that preserve their autonomy and independence. Falls are, in this sense, among the first causes of loss of autonomy, constituting a serious public health problem due to the clinical, social and economic consequences they have both on the person and on his/her family^1^.

Falls are the result of a complex interaction between multiple risk factors^2^, both intrinsic (biological and behavioral) and extrinsic (socioeconomic and environmental). Although some of these factors are not modifiable (age and chronic medical conditions), most of them are (lighting, soil surface, etc.). A recent systematic review^3^ shows that the development of intervention programs, although focusing only on one risk factor, reduces the occurrence of these adverse events and their consequences.

In the last decades, the scientific community is making great efforts at identifying risk factors for falls, however, with little attention being paid to risk assessment and documentation^4^ ^-^ ^5^. 

On the other hand, the current focus on aging is that it is active (independent, security in economic and social terms and integration into the community life). Action plans are being developed from the European Union to promote active aging through synergies between countries^6^. In this sense, with the focus on supporting long-term sustainability and efficiency of health systems, it is of interest to know how falls are addressed by health professionals. Thus, the objective of this study is to perform a diagnosis of the situation on documentation of falls and risk of falls in people older than 75 years in basic health units in Spain and Portugal.

## Methods

A mixed exploratory study was carried out in two stages: (i) qualitative descriptive and (ii) qualitative in basic health units (BHU) of two cities, one in southern Spain and the other in northern Portugal. In both cases, BHU constitute the minimum structure to guarantee the delivery of primary health care.

In the first stage of the study, the records of the health information systems of people aged 75 years and older documented in 2013 in Spain and in 2014 in Portugal were used as study objects. To determine the number of records, a sample size calculation was performed according to the following parameters: accuracy ± 5; 50% (situation of maximum uncertainty; no benchmark was established); confidence level of 95% and 10% as an unanticipated response percentage.

The value in the case of Spain was carried out by means of a two stages sample, first determining at random four BHU, and in these units, according to the population distribution of each one, the records were randomly selected. In this sense, the number of records included was 197.

In the case of Portugal, a systematic random sample of the total of records of people over 75 years of age from 11 BHUs belonging to the *Agrupamentos de Centros de Saúde Porto Oriental* (ACES) was used for sample selection. In total, a sample of 400 records was studied.

The statistical program to obtain the sample was Ene 3.0 (free distribution software). The source of the population used was, in the case of Spain, the Diraya program (electronic clinical history of the Public Health System of Andalusia); while for Portugal SClínico (evolutionary information system common to all health care providers and patient centered) was used.

In the qualitative step, the subjects of the study were healthcare professionals of the BHU included in the quantitative study. These subjects were, according to the structure of the BHU, mainly nursing and medical professionals. Sampling was intentional, selected by institutional intermediaries, and determined by data saturation. Thus, the number of health professionals in Spain was 16 (8 nurses and 8 doctors), while in Portugal, an intentional sampling was also carried out among nurses, totaling 56 professionals. The lower number of health professionals in Spain was due to the simultaneous use of the questionnaire with focus groups^7^; while in Portugal only questionnaires were used.

All manuscript authors, with experience in qualitative studies, participated in data collection at all stages. In the quantitative descriptive step, information was obtained from the clinical records of 2013 in Spain and 2014 in Portugal, of people over 75 years of age selected. This data was obtained during the year 2015. The data collected from the records were: sociodemographic variables (age, sex, schooling, marital status, company), clinical variables (medical history, number of drugs, drug types, weight, height, blood pressure, falls), risk factors for falls and nursing documentation, data that allowed characterizing the sociodemographic and clinical profile of the sample and perceive the relevance given by the health team to falls and risk of falls (evaluation, diagnosis and intervention).

In the qualitative phase, the data of the selected health professionals were obtained through a questionnaire divided into two parts: sociodemographic variables (age, sex, educational level and marital status), labor characteristics (working institution, length of service, professional category, specific training in the area of geriatrics/gerontology), and five open questions to know the perception of nursing professionals regarding the pertinence (or not) of integrating in their clinical practice the “risk of falls” of people aged 75 years and older, as well as identify possible obstacles that, in their opinion, could hamper the evaluation/intervention/ documentation of falls/fall risk. Before submitting the questionnaire, the professionals were informed about the objectives of the study and were given written information. All participants should have given their consent. The questions were as follows:

- In your opinion, as a health professional, is it your competence the assessment of risk of falling in the elderly? Why?
- Do you assess the falls/risk of falls that occur in the elderly? Why?
- In your opinion, does the health professional register falls/risk of falls in the elderly? Why?
- In your opinion, what kind of factors contribute to adherence/non-adherence to documentation of falls/risk of falls in the elderly?
- In your opinion, is the prevention of falls in the elderly an area of priority attention in primary health care? Why?

This information was collected between May and June 2015 in Spain and between March and May 2016 in Portugal. 

The analysis of the first step was performed through the software Statistical Package for the Social Sciences (IBM SPSS 22.0). The descriptive statistics developed implied the accomplishment of a previous Shapiro-Wilk normality test to prove the adjustment of the different variables to normality and thus to use parametric indexes or not. The sample was described by absolute and relative frequencies in qualitative variables, and central tendency (mean/median) and dispersion (typical deviation/interquartile range) in quantitative variables. Bivariate or simple non-parametric analyzes (Fisher’s test, U-Mann-Whitney) were performed to compare between groups, since the sample size for the study variables was lower than 30. For the hypothesis contrast statistical tests it was assumed a statistical significance with p<0.05 and confidence intervals with 95% confidence.

The data of the qualitative step were analyzed through Bardin’s analysis of thematic content (Bardin, 2009) to determine the “meaning cores” that form a message and whose presence or frequency can be significant for the analytical objective chosen^8^. In order to provide methodological rigor, the analysis was performed in three stages: (i) pre-analysis: reading of all the information to obtain a general information of the content; (ii) exploration of the material: coding information using “registration units” that establish a set of topics and a subsequent condensation of these themes into thematic categories; and (iii) treatment of results/ interpretation: presentation of the results in the form of summary tables that allow the interpretation and inference of the results with extracts from the “registration units”.

The data obtained from the two study scopes were compared by data triangulation, techniques, methods and researchers.

The study was approved by the Provincial Ethical Committees (Spain: Act no. 230 - ref. 2578; Portugal: nº 97/2014) of the study scopes for research on human subjects and all the processes were carried out in accordance with the 1964 Declaration of Helsinki and its subsequent modifications concerning the relevant ethical standards.

## Results

The selected records show similar populations regarding gender, with a majority of women (Spain: 64.5%, Portugal: 61.7%), and age (Spain: 81.3±4.7 years, Portugal: 82.6±5.3 years). The sociodemographic characteristics, number of drugs and the occurrence of falls are specified in Table 1.

**Table 1:** Sociodemographic characteristics, number of medicines and occurrence of falls. Cordoba, Andalusia, Spain, 2013; Porto, Portugal, 2014

Variables	Spain	Portugal
Gender		
Women	64.5% (127)	61.7% (246)
Men	35.5% (70)	38.3% (153)
Education		
None	5% (10)	5% (20)
Primary	11.7% (23)	35.5% (142)
Secondary	0% (0)	2.7% (11)
Higher	0.5% (1)	4% (16)
No record	82.8% (163)	52.8% (211)
Marital status		
Single	2% (4)	0.5% (2)
Married	14.3% (28)	23.7% (94)
Divorced	0% (0)	0.2% (1)
Widower	16.2% (32)	7.1% (28)
No record	67.5% (133)	68.5% (272)
Type of family		
Unitary	4.6% (9)	4% (16)
Nuclear	1.5% (3)	8% (32)
Single parent	2.6% (5)	2.2% (9)
Other	4.6% (9)	6.3% (25)
No record	80.7% (159)	79.5% (318)
N° of medicines		
None	6.6% (13)	14.6% (58)
< 5	27.9% (55)	27.4% (109)
≥ 5	65.5% (129)	53.5% (214)
Falls		
Yes	12.7% (25)	4.9% (19)
No	0% (0)	6.2% (24)
No record	87.3% (172)	88.9% (344)

Regarding the clinical characteristics, the body mass index (BMI) shows a Spanish population with light obesity (N=72, BMI=31.2±4.7 kg/cm^2^) and the Portuguese with overweight (N=260; BMI=26.3±4.6 kg/cm^2^). The systolic and diastolic blood pressure indexes were similar [Spain (N=162): 140.3±18.1 and 73.4±11.3 mmHg; Portugal (N=309): 137±13.9 and 73.7±8.5 mmHg].

The Spanish registries show as the most prescribed medications anticoagulant/antithrombotic drugs (50.3%), beta blockers (22.8%) and other antihypertensive drugs (67.5%), dyslipidemia drugs (38.1%), oral antidiabetics, (25,4%), diuretics (26.4%), antidepressants (20.8%) and anxiolytics/sedatives (19.3%). Portuguese registries for prescribed medication provided similar data: anticoagulants/antithrombotic drugs (27.2%), beta-blockers (16.9%) and other antihypertensive drugs (57.5%), dyslipidemia drugs (48.7%), oral antidiabetics (20%), diuretics (43.7%), antidepressants (17.2%) and anxiolytics/sedatives (35.8%).

The most frequent clinical entities in the Spanish registries were: hypertension (65.5%), osteoarticular diseases (44.7%), heart failure (32.5%), dyslipidemia (31%) and diabetes mellitus (29.4%). In the case of Portugal were: hypertension (66.2%), osteoarticular diseases (46.3%), diabetes (27%) and ophthalmic diseases (20.8%).

The gait aid were used in reduced cases (Spain: 13.2%, Portugal: 5.5%). In Portugal, unlike the Spanish registers, there were ethical habits, in fact, the percentage was quite high (64.5%). The characteristics of the fall and the nursing documentation included in the records are summarized in Table 2.

**Table 2:** Fall characteristics and nursing documentation. Cordoba, Andalusia, Spain, 2013; Porto, Portugal, 2014

Variables	Spain	Portugal
Injuries resulting from falls		
None	0% (0)	10.6% (2)
Fracture	20% (5)	31.5% (6)
Excoriation	8% (2)	15.8% (3)
Bruise	36% (9)	10.6% (2)
Laceration	24% 6)	0% (0)
Alteration of consciousness	12% (3)	5.2% (1)
No record	0% (0)	26.3% (5)
Place of fall		
Home	24% (6)	21.0% (4)
Street	56% (14)	0% (0)
Other	0% (0)	5.2% (1)
No record	20% (5)	73.7% (14)
Fall risk assessment		
Yes	29.9% (59)	2.1% (8)
No	0% (0)	0.4% (1)
No record	70.1% (138)	97.5% (391)
Assessment instrument		
Yes	5.6% (11)	1.8% (7)
No	24.4% (48)	0% (0)
No record	70% (138)	98.2% (393)
Nursing diagnosis for fall risk		
Yes	12.7% (25)	1.3% (5)
No	11.7% (23)	0% (0)
No record	75.6% (149)	98.7% (395)
Nursing diagnosis for fall		
Yes	3% (6)	0.2% (1)
No	9.6% (19)	0.8% (3)
No record	89.4% (176)	99% (396)

Regarding Portuguese registries, there was a significant association between the occurrence of falls and age (p=0.03, 85 *versus* 82 years), presence of dementia (p=0.04, 27.7% *vs.* 10.7%), presence of osteoarticular disease (p=0.01, 76.4% *vs*. 44.6%), and anti-vertiginous consumption (p=0.04, 27.7% *vs.* 10.8%); whereas, in the Spanish registries, statistical significance was verified with the presence of previous falls (p=0.02, 23.4% *vs*. 7.5%).

In both scopes, the characteristics of the falls were not systematically recorded. Regarding the time of the fall, no data was provided in the Portuguese context, while in Spanish, 80% of these provided this variable, being the morning (07:00-12:00h) the period of greatest occurrence of falls (40%).

The qualitative analysis focused on the problems of falls documentation, the evaluation of falls and the risk of falls. In this sense, the semi-structured interviews conducted with the health professionals allowed to obtain a diagnosis of the situation regarding documentation of falls and the risk of falls in the elderly. The sociodemographic, academic and professional characteristics in the two study scopes are shown in Table 3.

**Table 3:** Sociodemographic, academic and professional characteristics of the interviewees. Cordoba, Andalusia, Spain, 2015; Porto, Portugal, 2016

Variables	Spain (N=16)	Portugal (N=56)
Gender (N)	Women (10) Men (6)	Women (44) Men (12)
Age (Range)	39-61 years	31-55 years
Level of education (N)	Diploma/Degree (14) Master’s Degree (1) No record (1)	Graduation (47) Master’s Degree (9)
Marital status (N)	Married (10) Divorced (2) Single (3) No record (1)	Married (39) Divorced (4) Single (10) Married couple (3)
Time working on the current institution	22-388 months	2-246 months
Professional exercise time	5-39 years	6-31 years
Professional category	Doctors (9) Nurses (7)	Nurses (56)
Specialized in geriatrics/gerontology	Yes (2) No (14)	Yes (5) No (51)

The open answers to determine the potential barriers in the evaluation and intervention of the falls/fall risk were variable, however, similar data were obtained in both scopes. More than two-thirds of respondents in both scopes (Spain: 75%, Portugal: 90.2%) considered it their responsibility to assess the risk of falling of the elderly, justifying that it was an objective of their portfolio of services: *We have this evaluation as an objective of our Management Unit* (Spanish Health Professional- SHP3) and is ideally positioned to conduct the assessment: *We are the ones who are most in contact* (SHP5); *the same assessment should not be unique and exclusive to nursing assessment, the family doctor should also be considered, or most times contact the patient and carry out his/her evaluation* (Portuguese Health Professional - PHP11); even though several professionals considered that it was a nursing competence: *I consider that the family nurse, taking into account the holistic view that he/she has of his/her patients, have the competence to evaluate the risk of falling* (PHP12)*; we are (nursing staff) the professional group with greatest proximity to the patient / family, as well as with possibilities of evaluation in the residential area* (PHP53).

Specifically, the percentage of health professionals who reported assessing falls/risk of falls in their care practice was lower in Spain (56.3%) than in Portugal (84.4%). The professionals justified that it was within their competence: *it is the health professionals’ competence* (SHP4); to carry out preventive measures: *From here, to work towards the prevention of falls* (PHP9); *to adopt and provide the necessary ortho-prosthetic material (cane, walker, footwear, etc.)* (SHP7). Those who answered negatively considered that there were no appropriate scales for assessment, lack of time, or lack of relevance in relation to other care activities: *Due to the lack of appropriate scales and because there is no institutional protocol for fall risk assessment* (PHP10); *most of the time I assess the risk without registering it, because we consider other issues more relevant, as the treatment that we are going to carry out and that is more urgent* (PHP19); *most of the time due to lack of time to evaluate physical spaces* (PHP17).

In both contexts a large number of health professionals considered that falls / risk of falls were recorded (Spain: 75%, Portugal: 69.2). The explanations they provided were similar to the previous ones: lack of appropriate instruments and systems for registration or recent incorporation of service portfolio objectives and lack of time: *We have been recording it for two years* (SHP1); *due to the lack of appropriate scales. Because it is not a common practice or it is not common within the institution* (PHP10); *because our software is messy and very complex and the intervention is ambiguous* (PHP19); *it is often not detected, again due to the lack of time spent in the realization of households* (PHP17). On the other hand, the Spanish health professionals considered that this action was exclusively competent of the nursing staff: *the nursing does it* (SHP11).

Health professionals agreed that the factors that contributed to the registration / non-registration of falls were the lack of operability of the registration system, lack of time, awareness and motivation: *Complex computer system. Lack of time and work overload* (PHP18)*; professional awareness/motivation; work overload (no replacement); many nursing objectives in the contract program; high mean age of nursing staff makes computer work difficult* (SHP8)*; lack of awareness; lack of time; is not considered to influence their comprehensive care* (SHP1).

More than half of health professionals stated that falls prevention was a priority consideration (Spain: 56.2%, Portugal: 95.8%). *In primary care, primary prevention is our main goal. The falls imply an important health problem* (SHP2)*; I think it should be because falls are one of the main reasons that lead the elderly to lose autonomy in their activities of daily living and lead to major sequelae in terms of morbidity* (PHP6)*.* Nevertheless, those who responded negatively emphasized the morbidity and mortality of these events*: It is not a priority, but it is still important, since falls often leave the elderly incapacitated and this translates into a decrease in quality of life* (PHP17).

**Figure 1:**
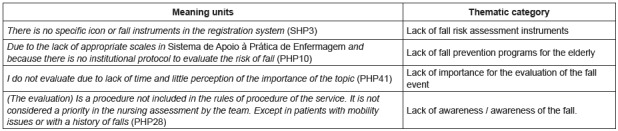
Emerging categories after data triangulation. Cordoba, Andalusia, Spain, 2015; Porto, Portugal, 2016

The inference and interpretation of the results, according to Figure 1, allows to establish emergent categories. Health professionals perceived that there was a lack of fall prevention programs in the elderly in terms of risk measurement instruments. As a consequence, practitioners reported that at the institutional level there was no effort to establish fall assessment protocols/measures, and there was therefore a lack of fall risk assessment. In fact, some health professionals perceived as not important the evaluation of the fall event. Finally, the institutional and professional situation about the fall event evidence low awareness.

The data triangulation obtained from both the analysis of the records of people over 75 years and from the semi-structured interviews to health professionals in both scopes, in different contexts and researchers, allowed to identify the following problems: (i) absence of fall risk records, due to the lack of adequate instruments, as informed by health professionals; (ii) lack of implementation of specific fall prevention programs, by undertaking individual and non-systematized measurements; (iii) lack of evaluation of falls and their characteristics, a fact contrasted with the professionals perception, the lack of records on the circumstances of falls, and the absence of preventive measures implemented after a fall event; (iv) lack of awareness about the fall event, a perception that was shared in both spheres by professionals and supported by the lack of institutional actions; and (v) underreporting of falls, showing a lack of registration culture at the level of health professionals and the institution itself. 

## Discussion

The present study provides evidence both quantitatively and qualitatively of the documentation problems of falls and risk of falls of people older than 75 years in basic health units in Spain and Portugal.

In both contexts, health professionals express that the fall is a problem of great relevance and that it is increasing due to the aging population. Nevertheless, in the studied registries it was shown the reduced number of documented falls. The causes that emerged from this absence of records were the lack of computer systems and friendly instruments that facilitate registration, work overload, as well as lack of awareness and motivation/awareness in assessing the risk of falls.

However, the quality of the recorded data will determine the success in patient safety^9^ ^-^ ^11^. In this line, several studies showed reasons similar to those raised in our study, of the causes of underreporting^12^ ^-^ ^14^. The main reasons given in these studies are: work overload, fear of guilt, the feeling that a report with quality guarantee was not performed, and the lack of knowledge by health professionals of the existence of the records or of registering. The authors’ recommendation is to modify the “blame” culture for one of freedom of registration, implementing user friendly information systems available to all professionals, as well as providing ongoing training on the uses and benefits of registration systems, as well as a deep analysis of the fall event and significant commitment of health professionals^15^. On the other hand, it should be clear that it is not so much about the number of records obtained that determines the registration system, but that a sufficient number is registered to build a safety idea^15^. This last author agrees with the perception of the Spanish and Portuguese health professionals of the present study, who report on the existence of events with serious injuries, but, on the other hand, there is no awareness that the system has a low registration rate, as it can be deduced from the fact that not many events with minor injuries or without consequences are included ^(^ ^14^.

Regarding the causes of fall, although no statistical significance was found in both contexts, the clinical records show that the majority of the falling elderly are female. Other studies also found a greater predisposition in females^16^, explaining this difference due to musculoskeletal and hormonal characteristics, as well as because they perform multiple tasks. In fact, the presence of osteoarticular diseases in people who fell was significantly more common (76.4%) than in those who did not suffer any falls (44.6%), a fact that also coincides with other similar series^16^. Therefore, it is essential to develop adjusted preventive measures, not only in relation to age - a significant variable in our study, but also to gender^17^. Statistical significance was also found for the presence of depression, consumption of antivertiginous medication and presence of previous falls. Recent studies of subjects living in communities also suggest that people over 80 years of age with vertigo, symptoms of depression, arthritis, who live alone and who have previously suffered falls are at increased risk of recurrences^18^ ^-^ ^20^.

However, the records studied in both contexts usually do not document the characteristics of the fall and social circumstances, variables that are of great importance to approach the fall appropriately^21^.

The qualitative phase of the study also emphasizes that the preventive approach in the elderly is addressed exclusively by the nursing staff. In both contexts, some health professionals did not perceive the fall risk approach as a primary and priority prevention, focusing exclusively on the treatment of the consequences of falls ^22^. In this sense, the nursing records related to the diagnosis and interventions were very scarce, evidencing a lack of awareness of the fall^23^. This is in accordance with similar series carried out in a hospital setting, where the prevalence of the diagnosis of risk of falls was 4%^24^. On the other hand, a study carried out by this research group^7^ highlights the lack of registration culture at the level of the health professional and the institution itself, which may be associated with the Iberian culture itself. Therefore, the development of studies regarding the cultural factor in the risk of falling in the elderly is necessary in future studies.

The triangulation of data allowed us to identify several problems that need to be addressed by primary care centers in order to be able to develop appropriate preventive measures, and thus, ensure patient safety. In a technological society such as today’s, it is necessary to develop user-friendly and accessible registration systems^24^. Moreover, there are also problems with the organization of services (lack of human resources, work overload, lack of protocols, etc.) and lack of awareness of the fall (lack of training on the problem, devaluation, lack of awareness, etc.), should be addressed through sensitization and training sessions directed at the entire multidisciplinary team.

Even if the study evidences the problem of documentation of falls and risk of falls in Spanish and Portuguese primary care centers, the results should be considered with caution when conducting the analytical study of the records retrospectively, since it is possible to lose data. On the other hand, the data obtained in the qualitative phase are subjective perceptions of health professionals, that is, at times they cannot always express their opinions^25^. Nevertheless, data saturation and triangulation decrease the possibility of bias in the results obtained.

## Conclusions

The falls in the elderly living in the community are perceived by Spanish and Portuguese health professionals as an area of priority attention. However, the assessment of the risk of falling is generally not considered, as being a consequence of organizational, logistical and motivational factors.

The problems identified emerge in a considerable extent due to lack of adherence to the registry, perceived in a similar way in two distinct areas of the Iberian Peninsula. In this sense, the cultural factor can be the common variable to the lack of record of the risk of falling and the circumstances of the fall.

Considering the nature of our study, it is necessary to take awareness measures and sensitize the multidisciplinary team of the primary care centers through training sessions, as well as carry out organizational modifications focused on protocols and computer systems of data collection.
